# [1,2-Bis(di­phenyl­phosphan­yl)ethane-κ^2^ *P*,*P*]chlorido­(isonicotinamide-κ*N*)palladium(II) nitrate aceto­nitrile monosolvate

**DOI:** 10.1107/S2414314621011718

**Published:** 2021-11-19

**Authors:** Rafael A. Adrian, Bradley J. Lagemann, Hadi D. Arman

**Affiliations:** a Department of Chemistry and Biochemistry, University of the Incarnate Word, San Antonio TX 78209, USA; b Department of Chemistry, The University of Texas at San Antonio, San Antonio TX 78249, USA; Purdue University, USA

**Keywords:** palladium, crystal structure, nitrate salt, hydrogen bond, coordinating chloride, dppe, isonicotinamide

## Abstract

The Pd^II^ central atom in the title complex, [PdCl(C_26_H_24_P_2_)(C_6_H_6_N_2_O)]NO_3_·CH_3_CN or [PdCl(dppe)(INAM)]NO_3_·CH_3_CN, where dppe is 1,2-bis­(di­phenyl­phosphan­yl)ethane and INAM is isonicotinamide, exists in a slightly distorted square-planar environment defined by the two P atoms of the dppe ligand, a chloride ligand and the N atom of the isonicotinamide pyridyl ring. The crystal packing in the structure is held together by hydrogen bonds between the amide of the INAM ligand and the nitrate ions that complete the outer coordination sphere. A mol­ecule of aceto­nitrile is also found in the asymmetric unit of the title complex.

## Abstract 2

The crystal structure of the title complex, [PdCl(dppe)(INAM)]NO_3_^.^CH_3_CN consists of a Pd^II^ metal center in a distorted square-planar environment with hydrogen-bond inter­actions contributing to the crystal packing. An aceto­nitrile mol­ecule completes the asymmetric unit.

## Graphical abstract

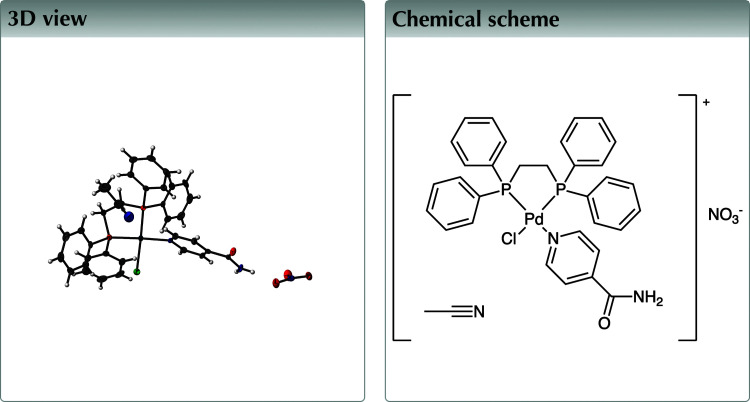

## Structure description

Palladium complexes containing 1,2-bis­(di­phenyl­phosphan­yl)ethane as a ligand have received much attention over the last decade because of their application in catalysis (Naghipour *et al.*, 2021 ▸; Thapa *et al.*, 2019 ▸). Recently, some of the focus has shifted to exploring their cytotoxicity (Cullinane *et al.*, 2018 ▸; Kuijpers & Blom, 2021 ▸) and biological activity (Al-Janabi *et al.*, 2021 ▸). In our research group, we have been exploring the synthesis of palladium(II) and copper(II) complexes containing various ancillary ligands and isonicotinamide as active ligand; isonicotinamide has proven to be an effective anti­metabolite due to its ability to enhance Sirt1 de­acetyl­ase activity, which reduces tumor growth (Li *et al.*, 2009 ▸). With that in mind, herein, we report the synthesis and structure of the title palladium(II) dppe complex.

**Figure 1:**
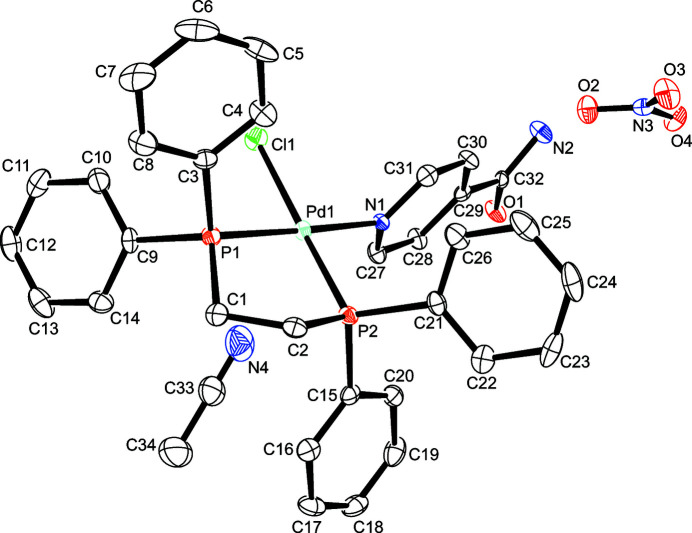
The structures of the mol­ecular entities of the title compound with displacement ellipsoids drawn at the 50% probability level; H atoms are omitted for clarity.

The asymmetric unit of the title compound, depicted in Fig. 1 ▸, consists of a Pd^II^ ion in a distorted square-planar coordination environment defined by the two phospho­rus atoms of the chelating dppe ligand, an N-bonded INAM mol­ecule, and a chloride ion. An aceto­nitrile mol­ecule and a nitrate ion complete the asymmetric unit. Selected bond lengths and angles involving the Pd^II^ atom are presented in Table 1 ▸. The Pd—Cl bond length in the title complex is in good agreement with the reported values of similar palladium(II) dppe complexes currently available in in the CSD (version 5.42 with update September 2021; Koide *et al.*, 1996 ▸; refcode TEPXIV; Owen *et al.*, 2002 ▸; refcode HUHZOZ; Owen *et al.*, 2003 ▸; refcode UMEDOF). Similarly, the Pd—N distance is also consistent with other structures found in the CSD, where a [Pd(dppe)]^2+^ unit is also bonded to the N-atom of a pyridyl ring (Guha *et al.*, 2012 ▸; refcode TIFYEO; Uehara *et al.*, 2013 ▸; refcode WINQOB; Mane *et al.*, 2021 ▸; refcode UTECEE). Nothing unusual is observed in the bond lengths and angles involving the dppe ligand.

**Figure 2:**
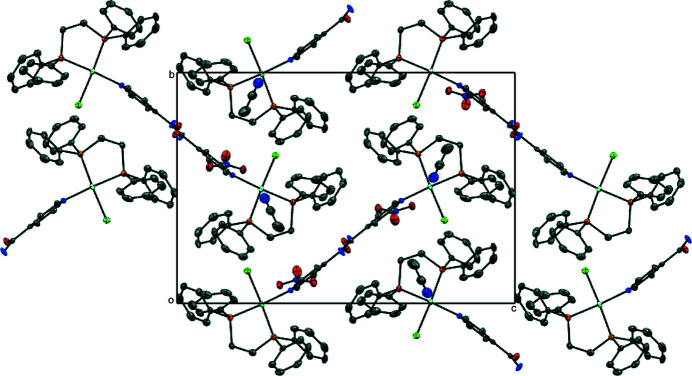
Perspective view of the packing structure of the title salt along the crystallographic *a*-axis; H atoms are omitted for clarity.

**Figure 3:**
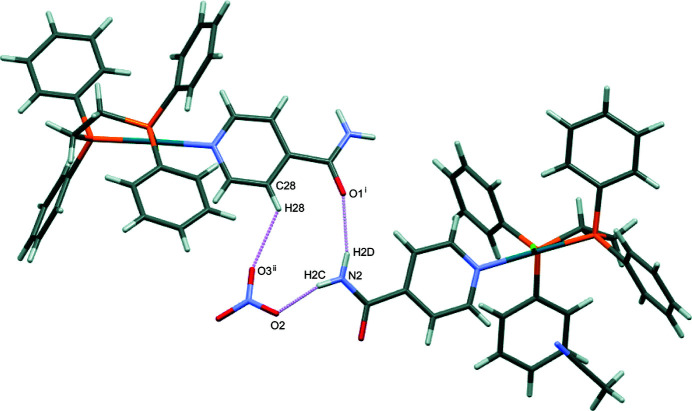
Capped sticks representation of the title compound showing the hydrogen-bond inter­actions (pink).

Several hydrogen-bonding motifs are present in the crystal structure, with numerical values collated in Table 2 ▸. In the crystal packing, mol­ecules self-assemble into sheets aligned along the *a* axis (Fig. 2 ▸) and are held together by N—H⋯O inter­actions between adjacent isonicotinamide ligands. The nitrate ions fill the void between the Pd^II^ complex ions inter­acting with the isonicotinamide ligands in different units through additional N—H⋯O and C—H⋯O inter­actions (Fig. 3 ▸).

## Synthesis and crystallization

To synthesize the title compound, [1,2-bis­(di­phenyl­phos­phan­yl)ethane]­dichlorido­palladium(II) (0.100 g, 0.174 mmol) was suspended in 40 ml of aceto­nitrile and stirred for 15 min. Solid AgNO_3_ (0.030 g, 0.18 mmol) was added to the suspension and heated with stirring at 303 K for 2 h. After removing AgCl by filtration, using a 0.45 mm PTFE syringe filter, the resulting pale yellow solution was used to grow crystals by vapor diffusion with diethyl ether at 278 K.

## Refinement

Crystal data, data collection and structure refinement details are summarized in Table 3 ▸.

## Supplementary Material

Crystal structure: contains datablock(s) I. DOI: 10.1107/S2414314621011718/zl4047sup1.cif

Structure factors: contains datablock(s) I. DOI: 10.1107/S2414314621011718/zl4047Isup2.hkl

Supporting information file. DOI: 10.1107/S2414314621011718/zl4047Isup3.mol

CCDC reference: 2120285

Additional supporting information:  crystallographic information; 3D view; checkCIF report

## Figures and Tables

**Table 1:** Selected geometric parameters (Å, °)

Pd1—Cl1	2.3564 (11)	Pd1—N1	2.100 (3)
Pd1—P1	2.2366 (11)	Pd1—P2	2.2577 (12)
			
P1—Pd1—Cl1	90.06 (4)	N1—Pd1—P1	176.86 (9)
P1—Pd1—P2	86.24 (4)	N1—Pd1—P2	96.81 (9)
N1—Pd1—Cl1	86.98 (9)	P2—Pd1—Cl1	173.44 (4)

**Table 2:** Hydrogen-bond geometry (Å, °)

*D*—H⋯*A*	*D*—H	H⋯*A*	*D*⋯*A*	*D*—H⋯*A*
N2—H2*C*⋯O2	0.88	2.04	2.891 (4)	163
N2—H2*D*⋯O1^i^	0.88	2.19	3.047 (4)	163
C28—H28⋯O3^ii^	0.95	2.39	3.082 (5)	129

Symmetry codes: (i) 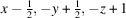 ; (ii) 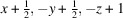 .

**Table 3:** Experimental details

Crystal data	
Chemical formula	[PdCl(C_26_H_24_P_2_)(C_6_H_6_N_2_O)]NO_3_·C_2_H_3_N
*M*_r_	765.43
Crystal system, space group	Orthorhombic, *P*2_1_2_1_2_1_
Temperature (K)	98
*a*, *b*, *c* (Å)	10.3343 (2), 14.8655 (4), 21.7942 (4)
*V* (Å^3^)	3348.12 (13)
*Z*	4
Radiation type	Mo *K*α
μ (mm^−1^)	0.77
Crystal size (mm)	0.30 × 0.10 × 0.03

Data collection	
Diffractometer	XtaLAB AFC12 (RCD3): Kappa single
Absorption correction	Multi-scan (*CrysAlis PRO*; Rigaku OD, 2019 ▸)
*T*_min_, *T*_max_	0.909, 1.000
No. of measured, independent and observed [*I* > 2σ(*I*)] reflections	36768, 6511, 5056
*R*_int_	0.054
(sin θ/λ)_max_ (Å^−1^)	0.616

Refinement	
*R*[*F* ^2^ > 2σ(*F* ^2^)], *wR*(*F* ^2^), *S*	0.027, 0.045, 0.97
No. of reflections	6511
No. of parameters	416
H-atom treatment	H-atom parameters constrained
Δρ_max_, Δρ_min_ (e Å^−3^)	0.60, −0.53
Absolute structure	Flack *x* determined using 1879 quotients [(*I* ^+^)−(*I* ^−^)]/[(*I* ^+^)+(*I* ^−^)] (Parsons *et al*., 2013 ▸)
Absolute structure parameter	−0.028 (12)

Computer programs: *CrysAlis PRO* (Rigaku OD, 2019 ▸), *olex2.solve* (Bourhis *et al.*, 2015 ▸), *SHELXL2014/7* (Sheldrick, 2015 ▸), and *OLEX2* (Dolomanov *et al.*, 2009 ▸).

## full crystallographic data

### Crystal data

**Table:**

[PdCl(C_26_H_24_P_2_)(C_6_H_6_N_2_O)]NO_3_·C_2_H_3_N	*D*_x_ = 1.518 Mg m^−^^3^
*M**_r_* = 765.43	Mo *K*α radiation, λ = 0.71073 Å
Orthorhombic, *P*2_1_2_1_2_1_	Cell parameters from 14630 reflections
*a* = 10.3343 (2) Å	θ = 2.6–28.4°
*b* = 14.8655 (4) Å	µ = 0.77 mm^−^^1^
*c* = 21.7942 (4) Å	*T* = 98 K
*V* = 3348.12 (13) Å^3^	Plank, clear colourless
*Z* = 4	0.3 × 0.1 × 0.03 mm
*F*(000) = 1560	

### Data collection

**Table:**

XtaLAB AFC12 (RCD3): Kappa single diffractometer	6511 independent reflections
Radiation source: Rotating-anode X-ray tube, Rigaku (Mo) X-ray Source	5056 reflections with *I* > 2σ(*I*)
Mirror monochromator	*R*_int_ = 0.054
ω scans	θ_max_ = 26.0°, θ_min_ = 2.3°
Absorption correction: multi-scan (CrysAlisPro; Rigaku OD, 2019)	*h* = −12→12
*T*_min_ = 0.909, *T*_max_ = 1.000	*k* = −17→18
36768 measured reflections	*l* = −21→26

### Refinement

**Table:**

Refinement on *F*^2^	Hydrogen site location: inferred from neighbouring sites
Least-squares matrix: full	H-atom parameters constrained
*R*[*F*^2^ > 2σ(*F*^2^)] = 0.027	*w* = 1/[σ^2^(*F*_o_^2^) + (0.015*P*)^2^ + 0.050*P*] where *P* = (*F*_o_^2^ + 2*F*_c_^2^)/3
*wR*(*F*^2^) = 0.045	(Δ/σ)_max_ = 0.001
*S* = 0.97	Δρ_max_ = 0.60 e Å^−^^3^
6511 reflections	Δρ_min_ = −0.53 e Å^−^^3^
416 parameters	Absolute structure: Flack *x* determined using 1879 quotients [(*I*^+^)-(*I*^-^)]/[(*I*^+^)+(*I*^-^)] (Parsons et al., 2013)
0 restraints	Absolute structure parameter: −0.028 (12)
Primary atom site location: iterative	

### Special details

**Table:**

Geometry. All esds (except the esd in the dihedral angle between two l.s. planes) are estimated using the full covariance matrix. The cell esds are taken into account individually in the estimation of esds in distances, angles and torsion angles; correlations between esds in cell parameters are only used when they are defined by crystal symmetry. An approximate (isotropic) treatment of cell esds is used for estimating esds involving l.s. planes.

### Fractional atomic coordinates and isotropic or equivalent isotropic displacement parameters (Å^2^)

**Table:**

	*x*	*y*	*z*	*U*_iso_*/*U*_eq_	
Pd1	0.67734 (2)	0.50517 (2)	0.74927 (2)	0.01367 (7)	
Cl1	0.68284 (10)	0.35931 (7)	0.79151 (5)	0.0218 (3)	
P1	0.64267 (10)	0.56282 (8)	0.84262 (5)	0.0151 (3)	
O1	0.9177 (2)	0.2660 (2)	0.49657 (13)	0.0188 (8)	
N1	0.7138 (3)	0.4446 (2)	0.66393 (15)	0.0130 (8)	
C1	0.6432 (4)	0.6856 (3)	0.83773 (18)	0.0174 (10)	
H1A	0.5924	0.7112	0.8720	0.021*	
H1B	0.7331	0.7082	0.8410	0.021*	
P2	0.64956 (9)	0.64655 (8)	0.71380 (5)	0.0143 (3)	
O2	0.8163 (3)	0.0909 (2)	0.40345 (13)	0.0285 (7)	
N2	0.7101 (3)	0.2228 (2)	0.48694 (15)	0.0197 (9)	
H2C	0.7284	0.1859	0.4566	0.024*	
H2D	0.6298	0.2279	0.5000	0.024*	
C2	0.5844 (4)	0.7147 (3)	0.77645 (18)	0.0168 (10)	
H2A	0.6042	0.7790	0.7690	0.020*	
H2B	0.4892	0.7079	0.7780	0.020*	
O3	0.6447 (2)	0.1373 (2)	0.35577 (13)	0.0347 (9)	
N3	0.7550 (3)	0.1013 (3)	0.35401 (17)	0.0213 (9)	
C3	0.4854 (3)	0.5284 (3)	0.87202 (18)	0.0172 (11)	
O4	0.8039 (3)	0.07869 (19)	0.30404 (13)	0.0251 (7)	
N4	0.9979 (4)	0.5452 (3)	0.7596 (2)	0.0487 (12)	
C4	0.4001 (3)	0.4843 (3)	0.83214 (19)	0.0230 (11)	
H4	0.4232	0.4750	0.7904	0.028*	
C5	0.2814 (4)	0.4542 (3)	0.8540 (2)	0.0298 (13)	
H5	0.2219	0.4262	0.8268	0.036*	
C6	0.2499 (4)	0.4647 (3)	0.9147 (2)	0.0352 (14)	
H6	0.1705	0.4412	0.9296	0.042*	
C7	0.3327 (4)	0.5092 (3)	0.95467 (19)	0.0346 (12)	
H7	0.3090	0.5180	0.9964	0.042*	
C8	0.4517 (4)	0.5410 (3)	0.9326 (2)	0.0266 (12)	
H8	0.5093	0.5714	0.9595	0.032*	
C9	0.7583 (4)	0.5316 (3)	0.90152 (18)	0.0171 (11)	
C10	0.7398 (4)	0.4525 (3)	0.93414 (19)	0.0250 (12)	
H10	0.6675	0.4153	0.9252	0.030*	
C11	0.8265 (4)	0.4273 (3)	0.97990 (18)	0.0276 (11)	
H11	0.8116	0.3741	1.0030	0.033*	
C12	0.9337 (4)	0.4795 (3)	0.9917 (2)	0.0285 (12)	
H12	0.9934	0.4621	1.0226	0.034*	
C13	0.9544 (4)	0.5575 (3)	0.9584 (2)	0.0278 (12)	
H13	1.0282	0.5936	0.9666	0.033*	
C14	0.8680 (4)	0.5832 (3)	0.91306 (19)	0.0219 (11)	
H14	0.8837	0.6362	0.8898	0.026*	
C15	0.8005 (4)	0.6961 (3)	0.68666 (18)	0.0165 (10)	
C16	0.8613 (4)	0.7658 (3)	0.71808 (19)	0.0201 (10)	
H16	0.8237	0.7905	0.7541	0.024*	
C17	0.9785 (4)	0.7988 (3)	0.6957 (2)	0.0242 (11)	
H17	1.0214	0.8460	0.7170	0.029*	
C18	1.0331 (4)	0.7638 (3)	0.6432 (2)	0.0256 (12)	
H18	1.1127	0.7872	0.6283	0.031*	
C19	0.9720 (4)	0.6942 (3)	0.6118 (2)	0.0271 (12)	
H19	1.0097	0.6702	0.5755	0.033*	
C20	0.8546 (4)	0.6597 (3)	0.63382 (18)	0.0199 (10)	
H20	0.8126	0.6119	0.6129	0.024*	
C21	0.5341 (4)	0.6623 (3)	0.65165 (18)	0.0165 (10)	
C22	0.5517 (4)	0.7282 (3)	0.60718 (19)	0.0222 (11)	
H22	0.6283	0.7636	0.6072	0.027*	
C23	0.4574 (4)	0.7425 (3)	0.5626 (2)	0.0281 (12)	
H23	0.4700	0.7867	0.5316	0.034*	
C24	0.3441 (4)	0.6914 (3)	0.5636 (2)	0.0324 (12)	
H24	0.2790	0.7014	0.5336	0.039*	
C25	0.3261 (4)	0.6268 (3)	0.6080 (2)	0.0304 (12)	
H25	0.2484	0.5926	0.6086	0.036*	
C26	0.4203 (4)	0.6111 (3)	0.6518 (2)	0.0227 (11)	
H26	0.4078	0.5657	0.6819	0.027*	
C27	0.8370 (4)	0.4304 (3)	0.64540 (17)	0.0177 (10)	
H27	0.9058	0.4587	0.6669	0.021*	
C28	0.8648 (4)	0.3759 (3)	0.59625 (18)	0.0161 (10)	
H28	0.9520	0.3684	0.5835	0.019*	
C29	0.7669 (3)	0.3321 (3)	0.56531 (18)	0.0132 (10)	
C30	0.6398 (3)	0.3463 (3)	0.58466 (18)	0.0163 (10)	
H30	0.5697	0.3176	0.5644	0.020*	
C31	0.6181 (4)	0.4027 (3)	0.63362 (18)	0.0169 (10)	
H31	0.5314	0.4125	0.6466	0.020*	
C32	0.8038 (4)	0.2709 (3)	0.51318 (18)	0.0127 (9)	
C33	1.0676 (5)	0.5989 (4)	0.7770 (2)	0.0360 (13)	
C34	1.1546 (5)	0.6688 (4)	0.8000 (2)	0.0596 (17)	
H34A	1.2106	0.6897	0.7666	0.089*	
H34B	1.2079	0.6443	0.8332	0.089*	
H34C	1.1034	0.7195	0.8156	0.089*	

### Atomic displacement parameters (Å^2^)

**Table:**

	*U*^11^	*U*^22^	*U*^33^	*U*^12^	*U*^13^	*U*^23^
Pd1	0.01207 (12)	0.01632 (15)	0.01261 (12)	0.00130 (15)	0.00071 (16)	−0.00188 (19)
Cl1	0.0249 (5)	0.0193 (6)	0.0211 (6)	0.0024 (5)	0.0007 (5)	0.0003 (5)
P1	0.0129 (6)	0.0181 (7)	0.0142 (6)	−0.0010 (5)	0.0009 (5)	−0.0010 (5)
O1	0.0074 (15)	0.028 (2)	0.0212 (17)	0.0013 (13)	0.0016 (12)	−0.0099 (15)
N1	0.0139 (19)	0.012 (2)	0.0130 (19)	−0.0015 (15)	−0.0022 (15)	−0.0006 (16)
C1	0.018 (2)	0.018 (2)	0.016 (2)	0.0007 (19)	0.0045 (19)	−0.003 (2)
P2	0.0125 (6)	0.0160 (7)	0.0144 (6)	−0.0015 (5)	0.0009 (5)	−0.0017 (5)
O2	0.0268 (17)	0.037 (2)	0.0217 (17)	0.0077 (17)	−0.0009 (16)	−0.0044 (16)
N2	0.013 (2)	0.027 (2)	0.019 (2)	−0.0015 (17)	0.0047 (15)	−0.0132 (18)
C2	0.0102 (19)	0.019 (3)	0.022 (2)	−0.0011 (18)	0.0025 (17)	−0.001 (2)
O3	0.0113 (16)	0.059 (2)	0.034 (2)	0.0073 (16)	0.0046 (14)	−0.0062 (18)
N3	0.021 (2)	0.018 (2)	0.024 (2)	−0.0038 (17)	0.0035 (19)	0.000 (2)
C3	0.013 (2)	0.017 (3)	0.021 (2)	−0.0008 (18)	0.0026 (18)	0.001 (2)
O4	0.0301 (17)	0.0287 (19)	0.0166 (16)	0.0077 (16)	0.0098 (15)	−0.0024 (15)
N4	0.053 (3)	0.046 (3)	0.047 (3)	0.008 (2)	−0.018 (3)	0.003 (3)
C4	0.017 (2)	0.025 (3)	0.027 (3)	0.003 (2)	0.0033 (18)	−0.008 (2)
C5	0.013 (2)	0.021 (3)	0.055 (4)	−0.001 (2)	0.000 (2)	−0.012 (3)
C6	0.014 (2)	0.027 (3)	0.064 (4)	−0.003 (2)	0.017 (2)	−0.004 (3)
C7	0.034 (3)	0.036 (3)	0.034 (3)	−0.002 (3)	0.024 (2)	−0.006 (3)
C8	0.025 (2)	0.027 (3)	0.028 (3)	−0.003 (2)	0.006 (2)	−0.008 (2)
C9	0.019 (2)	0.021 (3)	0.011 (2)	0.0050 (19)	−0.0020 (18)	−0.003 (2)
C10	0.027 (2)	0.026 (3)	0.023 (3)	−0.001 (2)	−0.005 (2)	−0.001 (3)
C11	0.038 (3)	0.025 (3)	0.019 (2)	0.008 (3)	0.004 (2)	0.004 (2)
C12	0.024 (2)	0.038 (4)	0.023 (3)	0.017 (2)	−0.009 (2)	−0.007 (3)
C13	0.015 (2)	0.036 (3)	0.032 (3)	0.005 (2)	−0.005 (2)	−0.013 (3)
C14	0.018 (2)	0.023 (3)	0.025 (3)	−0.002 (2)	0.003 (2)	−0.002 (2)
C15	0.014 (2)	0.017 (2)	0.018 (2)	0.002 (2)	−0.0025 (19)	0.005 (2)
C16	0.016 (2)	0.020 (3)	0.023 (3)	0.0024 (19)	0.000 (2)	0.002 (2)
C17	0.016 (2)	0.021 (3)	0.036 (3)	−0.004 (2)	−0.004 (2)	0.002 (2)
C18	0.016 (2)	0.029 (3)	0.032 (3)	−0.003 (2)	0.002 (2)	0.012 (3)
C19	0.021 (2)	0.042 (3)	0.019 (3)	0.005 (2)	0.005 (2)	0.007 (3)
C20	0.015 (2)	0.024 (3)	0.020 (2)	−0.002 (2)	−0.0030 (19)	0.002 (2)
C21	0.017 (2)	0.018 (3)	0.015 (2)	0.003 (2)	−0.0020 (19)	−0.005 (2)
C22	0.021 (2)	0.026 (3)	0.019 (3)	0.007 (2)	0.004 (2)	−0.002 (2)
C23	0.038 (3)	0.031 (3)	0.015 (3)	0.020 (3)	0.003 (2)	0.006 (2)
C24	0.028 (3)	0.041 (3)	0.028 (3)	0.016 (3)	−0.016 (2)	−0.011 (3)
C25	0.020 (2)	0.030 (3)	0.042 (3)	0.004 (3)	−0.011 (3)	−0.009 (3)
C26	0.021 (2)	0.018 (3)	0.029 (3)	0.004 (2)	−0.007 (2)	−0.002 (2)
C27	0.013 (2)	0.021 (2)	0.018 (2)	0.001 (2)	−0.0041 (19)	−0.001 (2)
C28	0.007 (2)	0.024 (3)	0.018 (2)	0.0019 (19)	0.0007 (18)	−0.003 (2)
C29	0.012 (2)	0.012 (3)	0.015 (2)	0.0029 (18)	−0.0002 (18)	0.004 (2)
C30	0.013 (2)	0.017 (3)	0.019 (2)	−0.0024 (19)	−0.0010 (18)	−0.004 (2)
C31	0.009 (2)	0.020 (3)	0.022 (3)	0.0045 (19)	−0.0003 (18)	0.003 (2)
C32	0.014 (2)	0.013 (2)	0.011 (2)	0.001 (2)	−0.0025 (19)	0.0012 (19)
C33	0.036 (3)	0.035 (3)	0.037 (3)	0.004 (3)	−0.011 (2)	0.004 (3)
C34	0.064 (4)	0.046 (4)	0.069 (4)	−0.014 (3)	−0.032 (3)	0.016 (3)

### Geometric parameters (Å, º)

**Table:**

Pd1—Cl1	2.3564 (11)	C11—C12	1.378 (6)
Pd1—P1	2.2366 (11)	C12—H12	0.9500
Pd1—N1	2.100 (3)	C12—C13	1.384 (6)
Pd1—P2	2.2577 (12)	C13—H13	0.9500
P1—C1	1.828 (4)	C13—C14	1.385 (6)
P1—C3	1.821 (4)	C14—H14	0.9500
P1—C9	1.814 (4)	C15—C16	1.392 (6)
O1—C32	1.233 (4)	C15—C20	1.391 (5)
N1—C27	1.352 (5)	C16—H16	0.9500
N1—C31	1.343 (5)	C16—C17	1.394 (5)
C1—H1A	0.9900	C17—H17	0.9500
C1—H1B	0.9900	C17—C18	1.379 (6)
C1—C2	1.530 (5)	C18—H18	0.9500
P2—C2	1.829 (4)	C18—C19	1.391 (6)
P2—C15	1.823 (4)	C19—H19	0.9500
P2—C21	1.820 (4)	C19—C20	1.402 (5)
O2—N3	1.259 (4)	C20—H20	0.9500
N2—H2C	0.8800	C21—C22	1.390 (6)
N2—H2D	0.8800	C21—C26	1.401 (5)
N2—C32	1.333 (5)	C22—H22	0.9500
C2—H2A	0.9900	C22—C23	1.394 (6)
C2—H2B	0.9900	C23—H23	0.9500
O3—N3	1.260 (4)	C23—C24	1.396 (6)
N3—O4	1.247 (4)	C24—H24	0.9500
C3—C4	1.401 (5)	C24—C25	1.376 (6)
C3—C8	1.378 (5)	C25—H25	0.9500
N4—C33	1.140 (6)	C25—C26	1.383 (5)
C4—H4	0.9500	C26—H26	0.9500
C4—C5	1.390 (5)	C27—H27	0.9500
C5—H5	0.9500	C27—C28	1.373 (5)
C5—C6	1.371 (6)	C28—H28	0.9500
C6—H6	0.9500	C28—C29	1.379 (5)
C6—C7	1.388 (6)	C29—C30	1.395 (5)
C7—H7	0.9500	C29—C32	1.505 (5)
C7—C8	1.403 (6)	C30—H30	0.9500
C8—H8	0.9500	C30—C31	1.376 (5)
C9—C10	1.387 (6)	C31—H31	0.9500
C9—C14	1.393 (5)	C33—C34	1.463 (7)
C10—H10	0.9500	C34—H34A	0.9800
C10—C11	1.391 (6)	C34—H34B	0.9800
C11—H11	0.9500	C34—H34C	0.9800
			
P1—Pd1—Cl1	90.06 (4)	C13—C12—H12	120.1
P1—Pd1—P2	86.24 (4)	C12—C13—H13	119.8
N1—Pd1—Cl1	86.98 (9)	C12—C13—C14	120.4 (4)
N1—Pd1—P1	176.86 (9)	C14—C13—H13	119.8
N1—Pd1—P2	96.81 (9)	C9—C14—H14	120.0
P2—Pd1—Cl1	173.44 (4)	C13—C14—C9	120.1 (4)
C1—P1—Pd1	109.20 (13)	C13—C14—H14	120.0
C3—P1—Pd1	110.82 (14)	C16—C15—P2	121.9 (3)
C3—P1—C1	107.71 (18)	C20—C15—P2	117.1 (3)
C9—P1—Pd1	116.11 (14)	C20—C15—C16	121.0 (4)
C9—P1—C1	107.14 (19)	C15—C16—H16	120.6
C9—P1—C3	105.48 (19)	C15—C16—C17	118.8 (4)
C27—N1—Pd1	120.0 (3)	C17—C16—H16	120.6
C31—N1—Pd1	120.1 (2)	C16—C17—H17	119.6
C31—N1—C27	118.3 (3)	C18—C17—C16	120.8 (4)
P1—C1—H1A	109.8	C18—C17—H17	119.6
P1—C1—H1B	109.8	C17—C18—H18	119.9
H1A—C1—H1B	108.2	C17—C18—C19	120.2 (4)
C2—C1—P1	109.4 (3)	C19—C18—H18	119.9
C2—C1—H1A	109.8	C18—C19—H19	120.1
C2—C1—H1B	109.8	C18—C19—C20	119.7 (4)
C2—P2—Pd1	107.89 (14)	C20—C19—H19	120.1
C15—P2—Pd1	112.25 (13)	C15—C20—C19	119.3 (4)
C15—P2—C2	109.45 (19)	C15—C20—H20	120.3
C21—P2—Pd1	117.25 (14)	C19—C20—H20	120.3
C21—P2—C2	104.05 (18)	C22—C21—P2	121.6 (3)
C21—P2—C15	105.49 (18)	C22—C21—C26	119.6 (4)
H2C—N2—H2D	120.0	C26—C21—P2	118.6 (3)
C32—N2—H2C	120.0	C21—C22—H22	119.9
C32—N2—H2D	120.0	C21—C22—C23	120.1 (4)
C1—C2—P2	110.4 (3)	C23—C22—H22	119.9
C1—C2—H2A	109.6	C22—C23—H23	120.3
C1—C2—H2B	109.6	C22—C23—C24	119.5 (4)
P2—C2—H2A	109.6	C24—C23—H23	120.3
P2—C2—H2B	109.6	C23—C24—H24	119.8
H2A—C2—H2B	108.1	C25—C24—C23	120.3 (4)
O2—N3—O3	118.8 (4)	C25—C24—H24	119.8
O4—N3—O2	120.7 (3)	C24—C25—H25	119.7
O4—N3—O3	120.5 (3)	C24—C25—C26	120.5 (4)
C4—C3—P1	118.4 (3)	C26—C25—H25	119.7
C8—C3—P1	121.6 (3)	C21—C26—H26	120.1
C8—C3—C4	119.9 (4)	C25—C26—C21	119.9 (4)
C3—C4—H4	120.2	C25—C26—H26	120.1
C5—C4—C3	119.5 (4)	N1—C27—H27	119.3
C5—C4—H4	120.2	N1—C27—C28	121.5 (4)
C4—C5—H5	119.9	C28—C27—H27	119.3
C6—C5—C4	120.3 (4)	C27—C28—H28	119.8
C6—C5—H5	119.9	C27—C28—C29	120.4 (4)
C5—C6—H6	119.6	C29—C28—H28	119.8
C5—C6—C7	120.9 (4)	C28—C29—C30	118.1 (4)
C7—C6—H6	119.6	C28—C29—C32	117.9 (3)
C6—C7—H7	120.5	C30—C29—C32	123.9 (4)
C6—C7—C8	119.1 (4)	C29—C30—H30	120.6
C8—C7—H7	120.5	C31—C30—C29	118.7 (4)
C3—C8—C7	120.3 (4)	C31—C30—H30	120.6
C3—C8—H8	119.9	N1—C31—C30	123.0 (4)
C7—C8—H8	119.9	N1—C31—H31	118.5
C10—C9—P1	119.3 (3)	C30—C31—H31	118.5
C10—C9—C14	119.1 (4)	O1—C32—N2	122.4 (3)
C14—C9—P1	121.5 (3)	O1—C32—C29	119.9 (4)
C9—C10—H10	119.8	N2—C32—C29	117.6 (3)
C9—C10—C11	120.5 (4)	N4—C33—C34	178.7 (6)
C11—C10—H10	119.8	C33—C34—H34A	109.5
C10—C11—H11	120.0	C33—C34—H34B	109.5
C12—C11—C10	120.0 (4)	C33—C34—H34C	109.5
C12—C11—H11	120.0	H34A—C34—H34B	109.5
C11—C12—H12	120.1	H34A—C34—H34C	109.5
C11—C12—C13	119.9 (4)	H34B—C34—H34C	109.5
			
Pd1—P1—C1—C2	34.8 (3)	C9—P1—C1—C2	161.4 (3)
Pd1—P1—C3—C4	−9.3 (4)	C9—P1—C3—C4	−135.7 (3)
Pd1—P1—C3—C8	167.7 (3)	C9—P1—C3—C8	41.3 (4)
Pd1—P1—C9—C10	−86.2 (3)	C9—C10—C11—C12	−2.1 (6)
Pd1—P1—C9—C14	91.1 (3)	C10—C9—C14—C13	−2.6 (6)
Pd1—N1—C27—C28	167.1 (3)	C10—C11—C12—C13	0.7 (6)
Pd1—N1—C31—C30	−166.1 (3)	C11—C12—C13—C14	−0.2 (6)
Pd1—P2—C2—C1	36.2 (3)	C12—C13—C14—C9	1.2 (6)
Pd1—P2—C15—C16	−109.9 (3)	C14—C9—C10—C11	3.1 (6)
Pd1—P2—C15—C20	68.4 (3)	C15—P2—C2—C1	−86.2 (3)
Pd1—P2—C21—C22	−147.5 (3)	C15—P2—C21—C22	−21.7 (4)
Pd1—P2—C21—C26	37.5 (4)	C15—P2—C21—C26	163.3 (3)
P1—C1—C2—P2	−45.1 (3)	C15—C16—C17—C18	0.6 (6)
P1—C3—C4—C5	177.5 (3)	C16—C15—C20—C19	−0.4 (6)
P1—C3—C8—C7	−176.4 (3)	C16—C17—C18—C19	−0.5 (7)
P1—C9—C10—C11	−179.5 (3)	C17—C18—C19—C20	−0.1 (7)
P1—C9—C14—C13	180.0 (3)	C18—C19—C20—C15	0.5 (6)
N1—C27—C28—C29	−1.7 (6)	C20—C15—C16—C17	−0.1 (6)
C1—P1—C3—C4	110.1 (3)	C21—P2—C2—C1	161.4 (3)
C1—P1—C3—C8	−72.9 (4)	C21—P2—C15—C16	121.3 (3)
C1—P1—C9—C10	151.5 (3)	C21—P2—C15—C20	−60.4 (4)
C1—P1—C9—C14	−31.2 (4)	C21—C22—C23—C24	1.3 (6)
P2—C15—C16—C17	178.1 (3)	C22—C21—C26—C25	−0.3 (6)
P2—C15—C20—C19	−178.7 (3)	C22—C23—C24—C25	−0.7 (7)
P2—C21—C22—C23	−175.8 (3)	C23—C24—C25—C26	−0.4 (7)
P2—C21—C26—C25	174.8 (3)	C24—C25—C26—C21	0.9 (6)
C2—P2—C15—C16	9.9 (4)	C26—C21—C22—C23	−0.8 (6)
C2—P2—C15—C20	−171.8 (3)	C27—N1—C31—C30	−0.1 (6)
C2—P2—C21—C22	93.5 (4)	C27—C28—C29—C30	1.3 (6)
C2—P2—C21—C26	−81.5 (3)	C27—C28—C29—C32	−178.0 (4)
C3—P1—C1—C2	−85.6 (3)	C28—C29—C30—C31	−0.3 (6)
C3—P1—C9—C10	36.9 (4)	C28—C29—C32—O1	−5.1 (6)
C3—P1—C9—C14	−145.8 (3)	C28—C29—C32—N2	174.3 (4)
C3—C4—C5—C6	−2.3 (7)	C29—C30—C31—N1	−0.3 (6)
C4—C3—C8—C7	0.6 (7)	C30—C29—C32—O1	175.8 (4)
C4—C5—C6—C7	3.2 (7)	C30—C29—C32—N2	−4.9 (6)
C5—C6—C7—C8	−2.1 (7)	C31—N1—C27—C28	1.1 (6)
C6—C7—C8—C3	0.2 (7)	C32—C29—C30—C31	178.9 (4)
C8—C3—C4—C5	0.5 (6)		

### Hydrogen-bond geometry (Å, º)

**Table:**

*D*—H···*A*	*D*—H	H···*A*	*D*···*A*	*D*—H···*A*
N2—H2*C*···O2	0.88	2.04	2.891 (4)	163
N2—H2*D*···O1^i^	0.88	2.19	3.047 (4)	163
C28—H28···O3^ii^	0.95	2.39	3.082 (5)	129

Symmetry codes: (i) *x*−1/2, −*y*+1/2, −*z*+1; (ii) *x*+1/2, −*y*+1/2, −*z*+1.
